# Interstate Air Pollution Governance in the United States: Exploring Clean Air Act Section 126

**DOI:** 10.1007/s00267-024-02002-3

**Published:** 2024-06-19

**Authors:** Alixandra Underwood, Richard Marcantonio, Danielle Wood, Paola Crippa

**Affiliations:** 1 International Food Policy Research Institute, 1201 Eye St., NW, Washington, DC 20005-3915 USA; 2 Kroc Institute for International Peace Studies in the Keough School of Global Affairs at the University of Notre Dame, 100 Jenkins Nanovic Hall, Notre Dame, IN 46556 USA; 3 Environmental Change Initiative at the University of Notre Dame, 700 Flanner Hall, Notre Dame, IN 46556 USA; 4 Department of Civil and Environmental Engineering and Earth Sciences at the University of Notre Dame, 162 Fitzpatrick Hall of Engineering, Notre Dame, IN 46556 USA

## Abstract

Air pollution is arguably the most pressing human health concern today, accounting for approximately 7–9 million premature deaths worldwide. In the United States, more than 40% of early deaths caused by air pollution are assessed to be caused by emissions produced by neighboring states. This article examines one of the governance mechanisms used by the U.S. to address this issue: section 126 of the Clean Air Act. Critical factors including case length, evidence used, and case outcome are compiled for the population of section 126 petitions submitted from 2000–2022. This evidence is assessed using comparative case analysis. The findings reinforce two issues with the petition process already identified in the literature–the use of cost as a proxy for significance and the excessive and unclear burden of proof placed on downwind states–adding texture to the latter issue by examining the modeling techniques used by downwind states. This analysis identifies lengthy response timelines as an additional issue and calls to attention the infrequency with which the EPA has formally accepted petitions. Collectively, these issues increase the cost, complexity, and unpredictability of filing a section 126 petition.

## Introduction

Air pollution is the single largest toxic pollution stream harming human health today (Fuller et al. 2022; Burnett et al. 2018; Pope et al. 2020; Vohra et al. 2021). Estimates vary, but some attribute at least 10 million premature deaths to air pollution from fossil fuel combustion alone (Vohra et al. 2021). Most of these premature deaths occur in low- and middle-income countries, but wealthy countries–especially low-income areas within wealthy countries–are also affected (Fuller, Sandilya, and Hanrahan 2019; Fuller et al. 2022; Yin et al. 2020; Burnett et al. 2018). In 2005, over 200,000 premature deaths per year were attributed to combustion emissions in the United States (Caiazzo et al. 2013). While there are many other forms of air pollutants, nitrogen oxide, ozone, and PM2.5 (airborne particles 2.5 microns in size or smaller) are the primary air pollutants driving premature mortality. In U.S. regulatory standards, these are listed as Criteria Air Pollutants (EPA, 2021).

Air pollution regulation presents many challenges. There is the challenge of stationary versus mobile air pollution sources, often categorized as point sources and nonpoint sources of air pollution (Lame and Marcantonio 2022). There is also the challenge of measuring or estimating the amount of pollution produced per emitting unit, whether mobile or not. Layered atop these is the challenge of jurisdictional authority and accountability (Lame and Marcantonio, 2022). Thus, the already difficult problem of air pollution regulation is exacerbated when crossing political boundaries, whether state (interstate) or national (transboundary).

In the United States, almost half of all premature deaths from air pollution are caused by emissions from neighboring states (Dedoussi et al. 2020). Interstate air pollution is not only of concern because of its prevalence, but also because evidence suggests intentionality. Findings from Helland and Whitford (2003), demonstrate an improbable rate of air pollution along border areas of states, suggesting that jurisdictional considerations are an important determinant of pollution incidence. These state level regulatory authority ‘spillovers’ capture the benefits of economic production while exporting the environmental costs to their neighbors and reducing the need for in-state regulatory enforcement (Konisky and Woods, 2010; Konisky and Woods, 2012). Thus, regulatory structures incentivize siting polluting facilities to border areas (Monogan et al., 2017). Such findings hold for both air and water pollution, further affirming the suspected intentionality of the practice (Sigman, 2005). These interstate air pollution challenges prompted the development of the Clean Air Interstate Rule (CAIR). As a predecessor to today’s Cross-State Air Pollution Rule (CSAPR), the CAIR had mixed results in implementation (Glasgow and Zhao, 2017). Recently, the EPA leveraged regulatory rulemaking from section 126 of the Clean Air Act to address interstate air pollution, including interstate transport programs and the “Good Neighbor” provision. Given the implications for alleged perpetrating states, these policies have been contentious (US EPA 2023b).

Using section 126 of the United States’ Clean Air Act as an example, this article illustrates the challenges of air quality management across political boundaries. It first explores the history of litigation regarding interstate air pollution in the US, then maps out the regulatory process for section 126. It then examines the relationship between various key factors and petition outcomes, using Maryland’s 2016 petition as a typical case. We take a deeper look at the modeling techniques used by downwind states and the EPA to prove emissions attribution, as well as the way model outputs are used in decision-making. The article concludes with a discussion about the illuminated shortcomings of section 126 and the importance of making it a more accessible tool for states burdened by upwind pollution and the implications for both interstate and transboundary pollution.

## Background and Literature Review

The Clean Air Act (CAA), first passed by Congress in 1963, is a comprehensive regulatory statute governing stationary and mobile air pollution sources in the United States. Among its many provisions, the CAA includes the establishment of the National Ambient Air Quality Standards (NAAQS) for specific air pollutants. Given the transitory nature of air pollution, a state or county’s ability to achieve its NAAQS is dependent not only on its own sources of emissions but also those from neighboring areas. In order to address this reality, the CAA includes two sections to manage upwind-downwind issues: section 110(a)(2)(D)(i)(I) (the ‘Good Neighbor’ provision) and section 126. There is an abundance of literature examining the effectiveness of the Good Neighbor Provision from a legal perspective, but very little addressing section 126 of the Clean Air Act. Luh’s (2021) “Being a Good Neighbor: Evaluating Federal Regulation of Interstate Air Pollution under the Cross-State Air Pollution Rule,” presents a recent history of section 126 petitions, as well as detailing the distinct regulatory niches of the Good Neighbor Provision and section 126.

The Good Neighbor Provision was a component of the CAA Amendments of 1970 (Clean Air Act Amendments (1970)). Initially requiring “intergovernmental cooperation,” the provision was reinforced in 1977 with stricter requirements for the inclusion of plans to address interstate transport in state implementation plans (SIPs), which are collections of state regulations to meet the NAAQS. Section 126 was part of the 1977 amendments to the CAA and includes a) the requirement that states provide written notice to all potentially affected states about “major proposed new (or modified) source[s],” b) a mechanism for states or political subdivisions to petition the EPA to find that a “source or group of stationary sources” violates the Good Neighbor Provision, and c) requirements for implicated sources, which must cease operation no more than “three months after such finding,” unless the EPA determines that the source can achieve compliance within three years (42 U.S.C. § 7426(b) 2023).

Section 126 was meant to fill a gap in the Good Neighbor Provision. In 1977, Congress pointed out the ineffectiveness of relying on upwind states to regulate interstate pollution when it is the downwind states that have the “incentive and need to act” (H.R. REP. No. 95-294, at 330, 1977). Therefore, section 126 provides an avenue for downwind states to take action by petitioning the EPA to directly regulate a source or group of sources, whereas the Good Neighbor Provision is a state-level remedy whereby the EPA initiates a call for SIPs from upwind states responsible for regulating the sources within their borders. This procedural difference between the Good Neighbor Provision and section 126 is one of three differences highlighted by Luh (2021). The second is a difference in regulated entities: states are regulated by the Good Neighbor Provision, whereas individual stationary sources are regulated by section 126. The third difference is in the EPA’s role. The Good Neighbor Provision only allows the EPA to directly regulate sources if a state has not adequately revised its SIP, at which point a federal implementation plan is required. In contrast, section 126 petitions call for direct regulation from the EPA, which is mandated to do so upon approval of the petition (42 U.S.C. § 7410(k)(5) 2023).

Another critical component of interstate air pollution governance in the U.S. is the various interstate transport programs that have been issued, including the Nitrous Oxide (NO_x_) Budget Trading Program in 2003 (EPA, 2023a); the Cross-State Air Pollution Rule (CSAPR), which was first enacted in 2015 (EPA, 2023b) and was amended three times (EPA, 2023c); and the recent Good Neighbor Plan, established in March 2023 to address the 2015 ozone NAAQS (EPA, 2024). Whereas the Good Neighbor Provision and section 126 are laws created by Congress, these programs are rules created by the EPA. They have included cap-and-trade, or emissions trading, of NO_x_, sulfur dioxide (SO_2_), and particulate matter (PM) allowances for between 19 and 28 states, primarily in the eastern United States. Each interstate transport program has faced litigation, sometimes from environmental groups calling for stricter regulation, as in *North Carolina vs. EPA* (North Carolina v. EPA, 2008), and sometimes from industry groups calling for more lenience, as in *EPA v. EME Homer City Generation, L.P*. (Kendall and Sweet 2014), with states and local governments also participating–sometimes calling for stricter and sometimes more lenient regulation.

The U.S. has made large strides in power sector emissions reductions since these interstate transport programs were first implemented. In 1990, five years before the first transport program (the Acid Rain Program) came into effect, the power sector emitted over six million tons of NO_x_ and 15 million tons of SO_2_ annually (LaCount et al., 2021). By 2019, after the CSAPR was implemented, annual power sector emissions had decreased to 877 thousand tons of NO_x_ and 969 thousand tons of SO_2_. Though greatly improved, these levels are still problematic, as demonstrated by a 2022 study by Mailloux et al. (2022), which found that 52,300 premature deaths in the U.S. could be prevented by efforts to eliminate energy-related emissions. This, coupled with Dedoussi et al.’s finding that 70% of early deaths from the electric power generation sector are caused by emissions from another state (2020), highlights a need for the U.S. to further improve its interstate air pollution governance.

The governance mechanisms discussed—the Good Neighbor provision, section 126, and interstate transport programs—influence one another in a regulatory ecosystem with feedback loops. The EPA accepts or denies section 126 petitions using the same four-part framework it uses when determining states’ Good Neighbor obligations for interstate transport programs (Federal Register, 2016). However, in the case of a section 126 petition, the petitioner (the downwind state) is responsible for satisfying each of the four steps in the framework (Federal Register, 2019). In the first step, downwind monitoring receptors expected to have difficulty meeting or maintaining NAAQS are identified (Federal Register, 2016). In step two, upwind states contributing at least one percent of the NAAQS to these receptors are identified. In step three, for these implicated states, upwind emissions that significantly contribute to downwind air quality problems are identified. For this, a multi-factor test is conducted, in which cost, downwind air quality impacts, and emissions reduction potential are assessed. This is essentially a cost-benefit analysis, for which the EPA has determined a cost threshold for each pollutant. For example, the threshold set in the CSAPR Update for the 2008 ozone NAAQS was $1,400 per ton. Finally, at step four, the EPA promulgates a federal implementation plan, which requires upwind states to adopt additional emissions reductions measures.

In analyzing interstate air pollution there is a fundamental complexity challenge such that, for a given area, how much pollution originates within a particular state versus a neighboring state must be determined (Farber, 2016). This also means the attribution of upwind emissions to a number of contributing upwind states must be delineated. This need for attributable delineation makes interstate air pollution regulation complex both technically and governmentally. The way the system is currently designed, though states are primarily focused on implementing the CAA within their borders, they must also evaluate their pollution contributions to downwind states and provide plans to reduce any significant contribution to downwind nonattainment areas. States may be less motivated to do the latter, as their constituents would more likely support emissions cuts to improve the air they breathe than to improve the air out-of-state, at potential economic costs. As recently as 2023, states failed to produce satisfactory transport SIPs. The EPA disapproved of the transport SIPs of 19 states, with modeling issues cited as a key factor in the disapproval determination of several states (Federal Register 2023).

Appendix W of Title 40 of the Code of Federal Regulations (CFR) Part 51, also known as the *Guideline on Air Quality Models*, specifies the modeling techniques accepted by the EPA (“preferred models”) and outlines requirements that “alternative models” must meet to be used for regulatory purposes (CAA, 2023). Additionally, when interstate transport programs are promulgated, the EPA has historically released technical support documents that provide modeling guidance applicable to states building a petition case. The most recent of these documents was released in 2023 alongside the Good Neighbor Plan (EPA, 2015). For the 2015 ozone NAAQS, the EPA recommends using the Weather Research and Forecasting (WRF) (Shamarock et al., 2008) model to generate meteorological fields required as input in a chemical transport model called CAMx (EVIRON, 2016).

Nonetheless, even with pollutant transport modeling that meets EPA standards and shows attribution, it is often insufficient to get a section 126 petition approved. This is because downwind states must also prove that there are cost-effective pollution abatement measures that would significantly reduce the air pollution they are receiving from upwind sources.

A key issue with the EPA’s regulatory framework that has been identified in previous investigations and is expanded upon here is that the burden of proof on downwind states filing section 126 petitions is both unclear and unmanageable (Luh 2021). The EPA expects petitioning states to conduct a global comparative analysis proving that cost-effective reductions can be made at each implicated source (*New York vs. EPA* 2020, line 1217). In its 2020 appeal of the EPA’s denial of its section 126 petition, the state of New York expressed the infeasibility of the EPA’s requirement that the petitioning state identifies “the current operating status of each named facility, the magnitude of emissions from each emitting unit within each named facility, the existing controls on each of these emissions units, additional control options on each emissions unit, the cost of each potential control option, the emissions reductions potential resulting from the installation of controls, and potential air quality impacts of emissions reductions (Federal Register, 2019).” Such analyses would require detailed inside information about each emitting facility’s operations and equipment. This information is often not publicly available, and sources charged with polluting are unlikely to willingly provide evidence that may lead to consequences for themselves (New York vs. EPA 2020). The difficulty (or impossibility) of conducting the required comparative analysis contradicts the intended purpose of section 126: to provide states with an additional, expedited avenue for interstate pollution reduction for NAAQS compliance.

Expanding on Luh’s findings (2021) regarding the excessive and unclear burden of proof placed on downwind states, this study contributes by exploring the relationship between key factors (see Table 1) and the outcomes of section 126 petitions. We examine the entire population of petitions filed from 2000 to 2022. In doing so, we are able to better understand the effectiveness of this management mechanism at supporting downwind states’ ability to meet and maintain NAAQS and, in turn, protecting human health. Of the key factors included in Table 1, we explore the modeling techniques used by states to determine emissions attribution in greater depth, as well as the EPA’s standards for accepting new techniques.

**Table 1:** Assessment of Section 126 Petitions Since 2000^a^

Petitioning State	Date Filed	Date of Final Action	Length of Case	Implicated Sources	Evidence Used	Evidence Generator	Result	EPA Reasoning
North Carolina	3/1/2004	4/28/2006	1 year, 2 months	EGUs in 13 states (Alabama, Georgia, Illinois, Indiana, Kentucky, Maryland, Michigan, Ohio, Pennsylvania, South Carolina, Tennessee, Virginia and West Virginia)	Regional Atmospheric Modeling System 3b Emissions Modeling System Urban-to-Regional Multiscale - One Atmosphere Model (URM-1ATM) Zero-Out Modeling Hybrid Single-Particle Lagrangian Integrated Trajectory (HYSPLIT)	Southern Appalachian Mountains Initiative (SAMI) EPA Ozone Transport Assessment Group (OTAG) North Carolina Division of Air Quality’s (NC DAQ)	Denied Emissions Trading Program (CAIR)	New FIPs and CAIR would address the problems cited in the petition
New Jersey	5/12/2010	No official response	NA	1 EGU in 1 state (Pennsylvania)	CALPUFF AERMOD (American Meteorological Society/Environmental Protection Agency Regulatory Model) version 07026 (model validation study to determine if CALPUFF produces more accurate predictions than AERMOD)	NJDEP EPA Cambridge Environmental Research Consultants ENSR Corp. TRC Environmental Corp.	NA	NA
New Jersey	9/17/2010	11/7/2011	1 year, 2 months	1 EGU in 1 state (Pennsylvania)	CALPUFF AERMOD (American Meteorological Society/Environmental Protection Agency Regulatory Model)	NJDEP EPA GenOn	Approved Increased regulation (emission limitations and compliance schedules for EGU)	EPA conducted an independent modeling analysis and found that the plant did violate the CAA
Connecticut	6/1/2016	4/13/2018	1 year, 10 months	1 EGU in 1 state (Pennsylvania)	Comprehensive Air Quality Model with extensions (CAMx) Ozone Source Apportionment Technology (OSAT)	Sonoma Technologies Inc. (STI) EPA	Denied	Insufficient evidence
Delaware	7/7/2016	10/5/2018	2 years, 3 months	1 EGU in 1 state (same EGU as Connecticut petition, in Pennsylvania)	CAMx OSAT	STI EPA	Denied Emissions Trading Program (CSAPR Update)	Existing programs, such as the CSAPR, would address the concerns in the petitions
Delaware	8/9/2016	10/5/2018	2 years, 2 months	1 EGU in 1 state (West Virginia)	CAMx OSAT	STI EPA	Denied Emissions Trading Program (CSAPR Update)	Existing programs, such as the CSAPR, would address the concerns in the petitions
Delaware	11/10/2016	10/5/2018	1 year, 11 months	1 EGU in 1 state (Pennsylvania)	CAMx OSAT	STI EPA	Denied Emissions Trading Program (CSAPR Update)	Existing programs, such as the CSAPR, would address the concerns in the petitions
Maryland	11/16/2016	10/5/2018	1 year, 11 months	36 EGUs in 5 states (Indiana, Kentucky, Ohio, Pennsylvania, and West Virginia)	Sparse Matrix Operator Kernel Emissions (SMOKE) Modeling System Weather Research and Forecasting (WRF) version 3.4 CAMx version 6.4	Maryland Department of the Environment STI EPA	Denied Emissions Trading Program (CSAPR Update)	Existing programs, such as the CSAPR, would address the concerns in the petitions
Delaware	11/28/2016	10/5/2018	1 year, 10 months	1 EGU in 1 state (Pennsylvania)	CAMx OSAT HYSPLIT	EPA Delaware Department of Natural Resources & Environmental Control (DNREC) STI	Denied Emissions Trading Program (CSAPR Update)	Existing programs, such as the CSAPR, would address the concerns in the petitions
New York	3/12/2018	10/18/2019	1 year, 7 months	350 sources in 9 states (Illinois, Indiana, Kentucky, Maryland, Michigan, Ohio, Pennsylvania, Virginia and West Virginia)	Eastern Regional Technical Advisory Committee (ERTAC) Community Multiscale Air Quality (CMAQ) WRF	EPA NY Department of Environmental Conservation Mid-Atlantic Regional Air Management Association (MARAMA)	Denied	Insufficient evidence; inadequate identification of additional cost-effective controls at sources; failure to identify relevant air quality problems

^a^The Federal Register includes EPA extensions of deadline for action on three additional section 126 petitions: one filed by Warrick County, IN, and the Town of Newburgh, IN, on 3/6/2008; one by the state of Delaware on 12/18/2008; and one by the town of Eliot, ME on 8/20/2013. These are not included in the table because there is no record of EPA final action in the register. Further investigation is required to determine whether the EPA dropped the petitions because the petitioners did not have the resources to sue the EPA and demand final action. A news article published one year after Eliot’s petition was filed says the EPA requested that Eliot consider withdrawing the petition, which the town rejected (https://www.seacoastonline.com/story/news/local/portsmouth-herald/2014/08/02/epa-should-help-not-hurt/36458357007/)

The information in this table comes from petition documents and EPA rules, mostly sourced from the Federal Register

## Methodology

### Information Sources

**Fig. 1:**
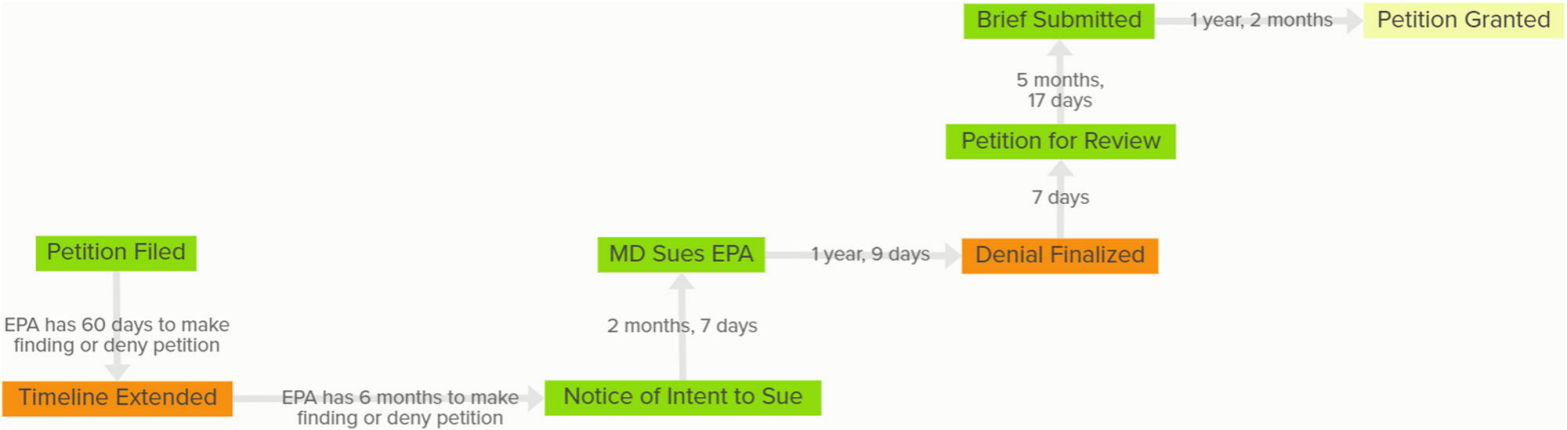
Maryland’s 2016 Section 126 Petition. This map displays the legal actions taken by the EPA (orange), Maryland (green), and the D.C. Circuit (yellow) following Maryland’s submission of a section 126 petition in November 2016. Original figure; Data Source: HLS Environmental and Energy Law Program

For the cases of petitions under Clean Air Act Part A Section 126, information on the EPA rules in the Code of Federal Regulations in the National Archives, specifically title 40, part 52, on *Approval and Promulgation of Implementation Plans*, was located and utilized to map the process. Critical factors of interest related to this research from the case descriptions included in these sources were extracted and are included in Table 1 and Fig. 1 (see below). Technical guidance from the EPA for modeling is also generally released in tandem with new interstate transport programs and is included and assessed here.

### Case Selection

To examine the efficacy of Clean Air Act Part A Section 126, we identified and documented the population of cases of petitions (see Table 1) from 2000–2022. This step helped us to understand the range of evidence, timelines, and outcome diversity in the cases.

For case comparisons (Table 1), critical independent factors included in the table were 1) petitioning state, 2) date filed, 3) date of final EPA action, 4) length of case, 5) implicated emitters (upwind stationary sources and states), 6) modeling techniques used as evidence, and 7) evidence source, meaning entities that generated the evidence. We also included critical dependent information in the table for the outcome/determination and the EPA rationale for the decision. These factors, distilled from case related records, were highlighted to provide for comparisons between the quality of petitions as well as outcomes across petitions. From this population of cases, we selected a typical case (Gerring et al. 2007) to represent the population of cases. Maryland was selected because it was similar to most petitions filed since 2000 with regard to actions taken by the state, the EPA, and the U.S. Court of Appeals for the D.C. Circuit. Despite this, it is influential as a case for the purpose of system mapping in that the D.C. Circuit ultimately granted, in part, Maryland’s petition for review of the EPA’s denial of its initial section 126 petition. In other words, Maryland’s petition went further than most in the legal system, though it was not ultimately approved by EPA.

### Process and Case Analysis

To illustrate and analyze the events surrounding Maryland’s 2016 section 126 petition, Kumu (2023) was used to develop the visualization of relationship maps (see Fig. 1). The interactive map^1^ produced and included in this article is publicly available on the Kumu platform included under the title Maryland Section 126 Petition.

The case comparison table (Table 1) was used to identify potential relationships between the variables, such as the type and source of the evidence-base for petitions and EPA determination. This step facilitated findings regarding information needs for Section 126 petition preparation, sufficiency of EPA guidelines for petitions, the role of modeling, and the relationship between determinations on petitions and the independent factors in the table, if any. Given the different contexts and circumstances of the petitions, we were not looking for causal homogeneity (Collier et al. 2004; Ragin 2004), but a general pattern. For pattern-matching (Yin, 2003), the expectation was a relationship between the quality of the evidence for the petition, with regard to EPA standards, and the outcome of the determination.

For the case analysis of the Maryland petition, the *Maryland Section 126 Petition* figure (Fig. 1) was used to visualize and detail case information to follow the Maryland petition from submission to decision. The combination of information made it possible to examine the movement of the petition through the process with relevant details of the evidence and the ruling. The analysis included examining challenges identified in the literature, such as the burden of proof on downwind states and lack of clarity from the EPA about what constitutes sufficient evidence; as well as highlighting new challenges, such as lengthy response timelines and the infrequency with which the EPA accepts petitions. This analysis allowed for systematic review of the process with regard to the evidentiary benchmarks for the petitioning state.

## Analysis and Discussion

The legal actions taken in response to Maryland’s 2016 section 126 petition are displayed in Fig. 1, and Table 1 lists all of the section 126 petitions filed since 2000 with the seven assessment variables introduced in the methods section.

The table reveals that only 13 petitions have been submitted since 2000, and only one of those was accepted by the EPA. Furthermore, the absence of certain states from Table 1 may be just as revealing as the petitions that have been filed. Recent investigations conducted in 2020 found that 50.90% of premature deaths caused by air pollution in Ohio in 2018 were from out of state emissions, yet Ohio has not submitted a section 126 petition since 2000 (Dedoussi et al. 2020). Given the magnitude of interstate air pollution and its health effects, this implies that Section 126 is not reaching its potential as a mechanism for providing relief to downwind states.

### A Typical Case: An Exploration of Maryland’s Petition Process

The 2016 Maryland petition illustrated in Fig. 1 implicated 36 coal plants in Indiana, Kentucky, Ohio, Pennsylvania, and West Virginia (EPA, 2018). According to CAA section 126(b), EPA has 60 days to either make a finding that the implicated sources do, indeed, violate the Good Neighbor Provision, or deny the petition. Determining that this was insufficient time to complete the review process, the EPA extended their timeline to respond by six months (EPA, 2018). This was not unique to Maryland’s case, as the EPA extended timelines to respond to North Carolina’s 2004 petition; Warrick County and Newburgh, Indiana’s 2008 petition; both of New Jersey’s 2010 petitions; Eliot, Maine’s 2013 petition; Connecticut’s 2016 petition; Delaware’s 2008 petition and all four of its 2016 petitions; and New York’s 2018 petition.

Maryland was not alone in suing the EPA when it failed to make a finding by the extended deadline–Delaware, Connecticut, and New York are among the other states that brought suits to the EPA in order to prompt a final action. For the petitions included in Table 1, the EPA took between 14 and 27 months from the date the petition was filed to make a final determination. Section 126 is meant to provide states an expedited avenue for obtaining relief from interstate pollution in order to comply with NAAQS, often under deadlines (H.R. REP. No. 95-294, at 331, 1977). This avenue is not satisfying its intended purpose, as the EPA consistently fails to meet its 60-day deadline, often only taking final action after being sued by the state.

The case of Maryland illustrates how slow response rates reduce the effectiveness of section 126. Maryland submitted its petition when the 2008 ozone NAAQS was in effect, however by the time the EPA took final action on the petition, the 2015 ozone NAAQS had come into effect. At the public hearing for the EPA’s denial of the petition, Maryland challenged the EPA’s assessment of the petition only for the 2008 NAAQS (Federal Register 2018). As the 2015 NAAQS are more stringent, Maryland asserted that the EPA’s analysis “necessarily demonstrate[d] that the named sources are also linked to the same monitor under the 2015 ozone standard.” The EPA responded that Maryland’s petition specifically requests the agency make a decision with regard to the 2008 NAAQS and that the EPA had not informed the public of any findings with regard to the 2015 NAAQS.

As with North Carolina and Delaware’s petitions, the EPA ultimately denied Maryland’s petition on the basis that an existing regulation addressed emissions from implicated facilities. This regulation was not in existence at the time the petition was filed, so this is a misnomer. One such “existing regulation” was the Cross-State Air Pollution Rule (CSAPR) Update, which was finalized in September 2016 (Federal Register 2016). Two of Delaware’s petitions were submitted earlier that year, which indicates the possibility that these petitions influenced the CSAPR Update. In fact, it is possible that this was Delaware’s intention, as information about the CSAPR Update was made publicly available in January 2015, when the EPA released a memo.

However, Maryland and Delaware’s follow-up actions suggest they were not satisfied with the CSAPR Update as the sole response to their petitions, as only seven days after the EPA finalized the denial, Maryland petitioned the D.C. Circuit to review the EPA’s decision (Federal Register 2017), and less than a month later, Delaware did the same (USCA 2018). In a brief submitted to the D.C. Circuit, Maryland said that “EPA’s reliance on the CSAPR Update to deny the petition was arbitrary and capricious,” the updated rule would not satisfy the Good Neighbor Provision, and the EPA could not use it to avoid the additional regulations proposed in the petition (USCA 2018).

The D.C. Circuit took almost as long to make a finding as the EPA had taken. Ultimately, it denied Delaware’s petition and granted Maryland’s in part, finding that the EPA was too quick to dismiss the state’s argument that four electric generating units (out of the 36 included in the petition) should be required to operate non-catalytic controls (Federal Register 2018). This finding came down to a question of cost-effectiveness: EPA stated in the CSAPR Update Rule that non-catalytic controls were not cost-effective (Federal Register 2016), but in *Wisconsin vs. EPA* the D.C. Circuit concluded that this statement was “impermissibly partial” and that a comparative cost-effectiveness analysis was in order (USCA 2019).

### Evidence Used: Modeling Techniques and Their Role in Decision-Making

The above examples again point to the issue that downwind states must not only prove that implicated sources in upwind states are contributing at least one percent of the NAAQS to receptors within their borders that are in danger of nonattainment–they must also prove that cost-effective reductions can be made at those sources. For the former, the EPA has made available technical support documents on acceptable modeling techniques, including in the text of the CAA itself, in Appendix W of Part 51 (CAA, 2023). However, for the latter, there are no easily accessible comprehensive guidelines for the multi-factor test–assessing cost, downwind air quality impacts, and emissions reduction potential–that the EPA expects to satisfy step three of their four-part framework.

Therefore, in order to assess the weight the EPA places on proof of pollution contribution versus cost-effectiveness, we examine the modeling techniques used by petitioning states in light of the EPA’s final action. If the EPA prioritizes proof of pollution contribution, we expect petitions to be approved for which the state used modeling techniques that a) closely follow the guidelines provided in the EPA’s technical support documents and/or b) produce relatively low error and bias, compared with the EPA’s interstate transport modeling.

The only section 126 petition since 2000 to have been officially approved by the EPA is New Jersey’s 2010 petition, which asked the EPA to find that an electric generating unit (EGU) in Pennsylvania was contributing to exceedances of the 1-hour sulfur dioxide (SO_2_) NAAQS in New Jersey (Martin, 2010). The New Jersey Department of Environmental Protection (NJDEP) used both the AERMOD (EPA, 2022) model recommended by the EPA for 1-hour SO_2_ at transport distances of less than 50 kilometers, and an alternative CALPUFF model (EPA, 2010). AERMOD assumes meteorological conditions do not change over the domain of interest for each unit of time considered (e.g., every hour) and that the transport and dispersion processes occur directly in the downwind direction of the emitting unit. Despite recent advances to the model, it is generally not accurate at distances larger than 50 km, as the assumption of uniformity and constant meteorological conditions is likely to be unrealistic. Conversely, CALPUFF, which is now included in the list of alternative models that the EPA suggests for more complex analyses or to quantify impacts at a larger distance (e.g., hundreds of kilometers), is able to account for spatio-temporal variability of meteorological conditions, as well as for chemical reactions in the atmosphere. Such a model is more computationally expensive than simpler alternatives, such as AERMOD.

Both models found that the impacts of the EGU were significantly higher than the NAAQS, so the EPA would have hypothetically approved the petition with either model. However, because the CALPUFF model showed far greater impacts, the use of this alternative model would have required a higher level of emissions reductions. Different meteorological inputs and model setups were key drivers of discrepancies in the predicted SO2 concentrations. The EPA cited concerns with the statistical model performance measures used by NJDEP, comparing them to the EPA’s 1992 Protocol for Determining the Best Performing Model (EPA, 1992).

To support its claim that “CALPUFF performed better and produced predictions of greater accuracy than AERMOD,” NJDEP’s petition included a previously-conducted model validation study of a nearby EGU (EPA, 2010). While this study provided evidence that CALPUFF performed better than AERMOD at one specific location in complex terrain, the EPA did not deem this result sufficient to consider the use of CALPUFF for the entire domain, as prior AERMOD evaluations at five complex terrain locations were cited to indicate a good performance of AERMOD.

This case demonstrates the importance of conducting a detailed model evaluation. If NJDEP had provided stronger evidence that CALPUFF performed better than AERMOD (i.e., model evaluation at multiple locations instead of just one), EPA might have considered the CALPUFF results credible, which could have led to more drastic emission controls. However, such a detailed model evaluation–providing more robust evidence of the harmful concentrations experienced and the results achievable with emission reduction policies in upwind states–may require acquiring additional in-situ observations. This is costly, time consuming, and without clarity of its impact on the outcome, further contributing to the already high burden of proof on downwind states (Luh, 2021).

Model uncertainty has important implications for policy decision-making (Alifa et al., 2022). In a limited-resource context, a model output that identifies an air pollution issue, but with very large uncertainty, may deserve less intervention priority than an issue identified with small uncertainty, as has already been highlighted in the context of climate change (Mastrandrea et al., 2010). Past research studies have highlighted challenges in interpreting traditional skill metrics (e.g., mean bias and means absolute error and their normalized versions) as they are affected by an asymmetry issue as the model overestimation is unbounded while underestimation is not, as well as by an inflation due to the occurrence of a few low concentration values relative to the whole observed dataset analyzed (Yu et al., 2006). Yu et al. (2006) proposed a range of new unbiased metrics to properly quantify model skills and measure the factor by which model results differ from observations and determine which observations should be used for such an assessment. For example, the relatively coarse grid spacing (i.e., 12 km) used in the EPA recommended modeling approach may be insufficient to capture local dynamic and chemical processes which may dictate the high spatial variability shown in the observations (Park et al., 2006). Therefore, estimating model performance based on the average concentration of the stations within the same grid cell is not necessarily representative of the actual model’s performance and may yield misleading conclusions of the model having lower biases than in reality (Park et al., 2006).

It should be noted that even the modeling techniques recommended by the EPA typically yield results with bias that is high relative to the pollution threshold for attribution (i.e., 1% of the NAAQS). The EPA Air Quality Modeling Final Rule Technical Support document of 2015 includes an evaluation of the EPA suggested modeling approach against monitoring sites that indicates that six of the U.S. climate regions have a mean bias within ± 5 ppb, while the Northern Rockies, the Northwest and the West have a mean bias larger than that, when considering days with a maximum daily average 8 hr ozone concentration > 60 ppb. While these numbers are aligned with the performance benchmark in Emery et al. (2017), they appear comparatively large if the attribution is based on 1% of the ozone standard (i.e., 0.75 ppb for an ozone limit of 75 ppb).

In 1995, Georgopoulos (1995) demonstrated the importance of identifying sources of modeling uncertainty and adopting stochastic models to define attainment and develop emission reduction strategies. Later work by Boylan et al. (2006) further discussed the need and challenges of setting a hard uncertainty threshold for PM, as various characteristics (e.g., concentration, composition, light extinction) could be used to assess model performance. The high level of uncertainty of even the EPA’s recommended modeling techniques indicates a need to invest in further model developments or in the use of more complex modeling tools. Sources of uncertainty in model output include model parameterizations (Crippa et al., 2019, Mallet and Sportisse, 2006), numerical approximations, and modeling choice (e.g. resolution, Crippa et al., 2017). It is important to consider the tradeoffs between these sources of uncertainty when considering adoption of transparent metrics/tools, in order to determine where to invest resources (i.e., decision-making, Alifa et al., 2022).

Returning to the case of Maryland, the state’s 2016 petition used modeling conducted by the University of Maryland, which consisted of the chemical transport model CAMx (EPA 2018). This model receives as input meteorological fields from WRF and emissions from the Sparse Matrix Operator Kernel Emissions (SMOKE) modeling system (Houyoux et al., 2000), which is recommended by the EPA. In the EPA’s final ruling on the petition, it found that Maryland provided sufficient evidence for steps one and two of the EPA’s four-step transport framework: 1) receptors within the state were in danger of nonattainment and 2) upwind sources contributed above one percent of the NAAQS to these receptors (Federal Register 2018). The EPA denied the petition at step 3, at which a multi-factor test must be conducted to assess the cost, potential for NO_x_ reduction, and downwind air quality effects of the proposed emissions reduction strategy.

Maryland’s petition provided evidence that the installed NO_x_ control equipment at the analyzed EGUs were not optimally operated, resulting in higher NO_x_ emissions deemed responsible for ozone exceedances experienced in Maryland (EPA 2018). Maryland’s petition focused on ensuring that more controls were run at the units during the ozone season, to prevent higher NO_x_ emissions, and urged the EPA to implement regulations on control practices. The EPA did not find that Maryland’s petition was technically deficient, but rather finalized the denial based on an independent assessment run by the EPA, from which they concluded there were no additional cost-effective reductions for the EGUs named in the petition (Federal Register 2018). According to this assessment, the emissions reduction strategy proposed by Maryland would have cost $3400 per ton of NOx reduced, and, as laid out in the CSAPR Update, a control strategy is only deemed cost effective if its marginal cost is no more than $1,400 per ton.

The preceding analyses of the New Jersey and Maryland cases indicate that a) the EPA has high standards for accepting anything other than their recommended modeling techniques, with the expectation that states not only prove the new model is adequate, but also that the recommended model is inadequate; and b) high-quality evidence of the effects of upwind source emissions is not enough for a petition to be accepted, as the EPA’s multi-factor cost-effectiveness assessment ultimately determines the fate of a petition.

## Implications

Previous studies illuminate several issues with section 126 as a mechanism to provide timely relief to downwind states, including the use of cost as a proxy for significance and the excessive and unclear burden of proof placed on downwind states. This study adds texture to the latter issue by examining the modeling techniques used by downwind states and the EPA’s analysis of those techniques. We also bring lengthy response timelines to the forefront of the discussion. Collectively, these issues increase the cost, complexity, and unpredictability of filing a section 126 petition. States may not have or be willing to use the time, money, and personnel necessary to create a section 126 petition and take the follow-up legal actions that are often required (suing the EPA and taking the case to the DC Circuit), especially given the uncertainty of the outcome and the infrequency with which the EPA has historically accepted petitions.

This study is unique in that it compiles every section 126 petition filed since 2000, allowing for a comprehensive comparative case analysis, and provides a visualization of the legal actions taken in a typical petition case. Findings from the review of this population of cases–including the limited number of petitions submitted and the fact that only one of them was officially accepted by the EPA–has implications for the sufficiency of section 126 as a mechanism for downwind states to satisfy the NAAQS and protect human health. We also find that the EPA has consistently failed to meet section 126 deadlines, limiting the effectiveness of the mechanism in providing timely relief in order that downwind states might meet NAAQS under deadlines. The table and figure provided here may be useful for future studies, especially the former, as a complete compilation of cases and their outcomes does not exist elsewhere. These resources are particularly relevant given the recent passage of the Good Neighbor Plan.

### Burden of Proof and Cost-Effectiveness

This study illustrates the substantial proof required to use a modeling technique other than those recommended by the EPA. This burden, placed on downwind states that may find the EPA’s modeling results unsatisfactory, would be eased if the state only had to prove that the new model is contextually adequate, not that the EPA’s recommended model is inadequate, which requires running costly model evaluations at multiple locations.

The New Jersey case illustrates how different models and model setups can lead to large discrepancies, thus posing the question of what information decision making should be based on. Currently, even the best models produce results with relatively high bias compared with the levels of pollution in question. As the uncertainty of a model’s outputs has implications for policy decision-making purposes (i.e., how much weight to place on the results and how much to prioritize the issue), an effective first step would be to invest more in modeling advancements generally.

However, as this and previous studies demonstrate, the question of modeling technique is moot if the downwind state is unable to identify a cost-effective method to reduce emissions. We emphasize Luh’s (2021) finding that, while an agency may analyze cost-effectiveness to select the pathway via which it will carry out a mandate, it is unlawful for the agency to choose not to regulate altogether on the grounds that it would be costly to do so (Federal Register, 1993). Section 126 may be a more just and feasible mechanism if downwind states were only responsible for the first two steps of the EPA’s four-part framework: a) identifying monitoring receptors within their borders in danger of nonattainment of NAAQS, and b) identifying stationary sources in upwind states that are contributing at least one percent of the NAAQS to those receptors.

### Lengthy Response Timelines

This study illustrates that a) the EPA has consistently failed to meet the 60-day deadline for making findings on section 126 petitions and b) this limits the effectiveness of the mechanism, as new interstate transport programs or NAAQS are often promulgated before the EPA makes a finding. The EPA has repeatedly used the former in their final responses to petitions, waiting until the transport program has been passed to deny the petition on the grounds that the new program will address the emissions implicated in the petition.

Potential reasons for the EPA’s consistent failure to meet the 60-day deadline, in addition to stalling until a new transport program is promulgated, may involve insufficient resources or personnel allocated to this issue. It may also involve a lack of political will or resistance and/or incentives from actors, such as corporations, that would be hurt by the stricter regulations proposed in section 126 petitions. If the former, insufficient resources, is the primary reason, the solution may be as straightforward as dedicating more resources to the EPA offices that review and respond to section 126 petitions. The latter, lack of political will, is a more complex problem, which may be entrenched in the larger governance systems in which the EPA operates.

This article helps build a foundation for a discussion around the effectiveness of section 126 of the CAA. To date, this discussion in the academic literature is surprisingly limited, given the fact that emissions from neighboring states cause almost half of all premature deaths from air pollution in the United States (Dedoussi et al. 2020). The EPA relies primarily on the Good Neighbor Provision (section 110(a)(2)(D)(i)(I) of the CAA) and interstate transport programs (the Good Neighbor Plan being the most recent) to govern this issue. However, as the only avenue for downwind states to participate in the governance of the upwind emissions that affect them, section 126 is a critical component of the EPA’s overall governance strategy. Together with Luh’s 2021 findings, our findings on the shortcomings of the petition process can be used for further research to identify paths forward to make this avenue more accessible to downwind states and more effective at providing equal protection from an undue burden of harmful air pollution.

## Footnotes

1 https://kumu.io/eci-air-pollution-governance/legal-cases#section-126-case
